# Mechanism of Association and Reciprocal Activation of Two GTPases

**DOI:** 10.1371/journal.pbio.0020320

**Published:** 2004-09-21

**Authors:** Shu-ou Shan, Robert M Stroud, Peter Walter

**Affiliations:** **1**Department of Biochemistry and Biophysics, University of CaliforniaSan Francisco, San Francisco, CaliforniaUnited States of America; **2**Howard Hughes Medical Institute, University of CaliforniaSan Francisco, CaliforniaUnited States of America

## Abstract

The signal recognition particle (SRP) mediates the cotranslational targeting of nascent proteins to the eukaryotic endoplasmic reticulum membrane or the bacterial plasma membrane. During this process, two GTPases, one in SRP and one in the SRP receptor (named Ffh and FtsY in bacteria, respectively), form a complex in which both proteins reciprocally activate the GTPase reaction of one another. Here, we explore by site-directed mutagenesis the role of 45 conserved surface residues in the Ffh-FtsY interaction. Mutations of a large number of residues at the interface impair complex formation, supporting the importance of an extensive interaction surface. Surprisingly, even after a stable complex is formed, single mutations in FtsY can block the activation of GTP hydrolysis in *both* active sites. Thus, activation requires conformational changes across the interface that coordinate the positioning of catalytic residues in both GTPase sites. A distinct class of mutants exhibits half-site reactivity and thus allows us to further uncouple the activation of individual GTPases. Our dissection of the activation process suggests discrete conformational stages during formation of the active SRP•SRP receptor complex. Each stage provides a potential control point in the targeting reaction at which regulation by additional components can be exerted, thus ensuring the binding and release of cargo at the appropriate time.

## Abstract 2

A molecular dissection of the bacterial signal recognition particle-receptor complex reveals discrete stages that provide control points in the protein targeting reaction, ensuring appropriate timing of binding and release

## Introduction

GTPases comprise a superfamily of proteins that provide molecular switches to regulate many cellular processes, including translation, signal transduction, cytoskeletal organization, vesicle transport, nuclear transport, and spindle assembly (Gilman 1987; Bourne et al. 1991). In many cases, the GTPases exert their regulatory function through a “GTPase switch” mechanism (Bourne et al. 1991) in which the GTPase assumes two alternative conformational states: an active, GTP-bound state and an inactive, GDP-bound state. Each state is kinetically stable, and interconversion between these states is facilitated by external regulatory factors, such as GTPase-activating proteins (GAPs) and guanine nucleotide exchange factors (GEFs).

Two homologous GTPases, one in the signal recognition particle (SRP) and one in the SRP receptor (SR; called Ffh and FtsY in bacteria, respectively), mediate the cotranslational targeting of membrane and secretory proteins to the eukaryotic endoplasmic reticulum (ER) membrane or the bacterial plasma membrane. During the targeting reaction, SRP and SR switch between different functional states (Walter and Johnson 1994; Keenan et al. 2001). SRP first binds to a nascent polypeptide that contains a signal sequence as it emerges from the ribosome (Walter et al. 1981; Pool et al. 2002). The ribosome•nascent chain complex (RNC) is then delivered to the membrane via an interaction between the GTP-bound forms of SRP and SR. Upon arrival at the membrane, SRP releases its “cargo,” the RNC, to the translocation apparatus or the translocon (Walter et al. 1981; Gilmore et al. 1982a, 1982b). Once the RNC is released, both SRP and SR hydrolyze their bound GTPs to drive dissociation of the SRP•SR complex, allowing the SRP and SR components to be recycled (Connolly and Gilmore 1989; Connolly et al. 1991). Analogous to other GTPases, the switch in the functional states of SRP and SR is coordinated by their GTPase cycles.

However, the regulatory mechanism of the SRP family GTPases provides a notable exception to the “GTPase switch” paradigm. Unlike many other GTPases, no external GEFs or GAPs are known for the SRP and SR GTPases. Instead, Ffh and FtsY bind nucleotides weakly, and nucleotide dissociation and exchange are very fast (Moser et al. 1997; Jagath et al. 1998; Peluso et al. 2001); thus, there is no requirement for external GEFs to facilitate their conversion from the GDP- to GTP-bound forms. In addition, Ffh and FtsY reciprocally activate each other's GTPase activity upon formation of the Ffh•FtsY complex (Powers and Walter 1995; Peluso et al. 2001); thus, there is no requirement for external GAPs to facilitate their conversion from the GTP- to GDP-bound forms.

The structure of Ffh and FtsY also defines them as a unique subgroup in the GTPase superfamily (Freymann et al. 1997; Montoya et al. 1997). Both proteins contain a central GTPase “G” domain that shares homology with the classical *Ras* GTPase fold. In addition, all SRP family GTPases contain a unique “N” domain, which together with the G domain forms a structural and functional unit called the NG domain. The crystal structures of the individual Ffh and FtsY NG domains show that both proteins have a wide-open GTP-binding pocket, and the apoforms of these proteins are stabilized by a network of side-chain interactions in the empty active site (Freymann et al. 1997; Montoya et al. 1997); the need to reposition the active-site residues for binding nucleotides may contribute to the low nucleotide affinities of these GTPases. Recently, the crystal structure of the GTP analog-bound Ffh•FtsY complex was determined (Egea et al. 2004; Focia et al. 2004). The two proteins form a pseudosymmetrical heterodimer via an extensive interaction surface that includes both the G and N domains. A composite active site is formed at the interface in which the two nucleotides are “twinned” in a head-to-tail manner, forming reciprocal hydrogen bonds between the ribose 3′-OH of one GTP and the γ-phosphate of the other. Hydrolysis of the nucleotide at each active site is also facilitated by multiple catalytic groups from its own protein, brought into the active site by conformational rearrangements that occur upon complex formation. These substrate-substrate interactions in *trans* and active site-substrate interactions in *cis* thus provide a novel mechanism for the GTP-dependent association and reciprocal activation between the two GTPases.

The unique structural and functional properties of the SRP and SR GTPases raise intriguing questions: (i) How do these GTPases act as reciprocal activating proteins for one another, and (ii) how does the SRP family of GTPases switch between the “on” and “off” states, as the GTPases are predominantly in the GTP-bound state as they enter the targeting cycle and no stable, GDP-bound state exists under cellular conditions?

In a previous paper, we described a scanning mutagenesis study of the conserved surface residues of Escherichia coli FtsY and showed that mutations that have deleterious effects on the Ffh-FtsY interaction define a large surface patch on FtsY that lies on its interaction surface with Ffh identified in the crystal structure (Egea et al. 2004). Here we show that these mutants can be categorized into distinct classes, each defective at a different step during the Ffh-FtsY interaction, suggesting that the Ffh-FtsY interaction is a dynamic process that involves multiple experimentally separable conformational changes. Thus, the mutants allow us to glean mechanistic insights into the alternative molecular switch that allows the SRP and SR to change their functional states.

## Results

**Figure 1:**
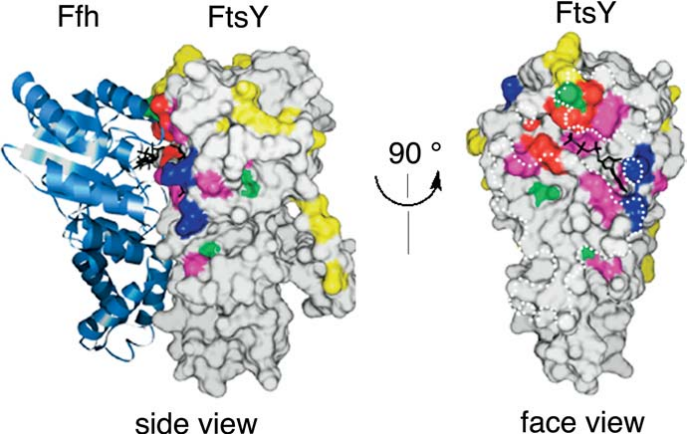
The Mutational Effects in E. coli FtsY Mapped onto the Crystal Structure of the Ffh•FtsY Complex The bound nucleotides are shown as black sticks, and the dotted white lines in the interface view outline the contact surface of Ffh with FtsY. The colors denote different classes of mutational effects: blue, Class I mutants defective in complex formation; red, Class II mutants defective in the reciprocal GTPase activation; magenta, Class III mutants defective in both steps; green, Class IV mutants exhibiting half-site reactivity; yellow, Class V or neutral mutants.

In light of the recently published structures of the Ffh•FtsY complex, kinetic analyses become increasingly valuable in unraveling the dynamic nature of the Ffh-FtsY interaction. To this end, we generated 45 site-directed mutants that were made in surface residues of FtsY. As previously described (Egea et al. 2004), all but one mutation that functionally compromise the Ffh-FtsY interaction map to the extensive interaction surface between the two proteins (Figure 1). As we show below, dissection of the mutational effect on individual steps allows us to divide the deleterious mutants into distinct classes: Class I mutants primarily affect complex formation, Class II mutants primarily affect the reciprocal GTPase activation, Class III mutants are defective in both steps, and Class IV or half-site mutants block the activation of only one GTPase site in the complex (Table 1).

**Table 1:**
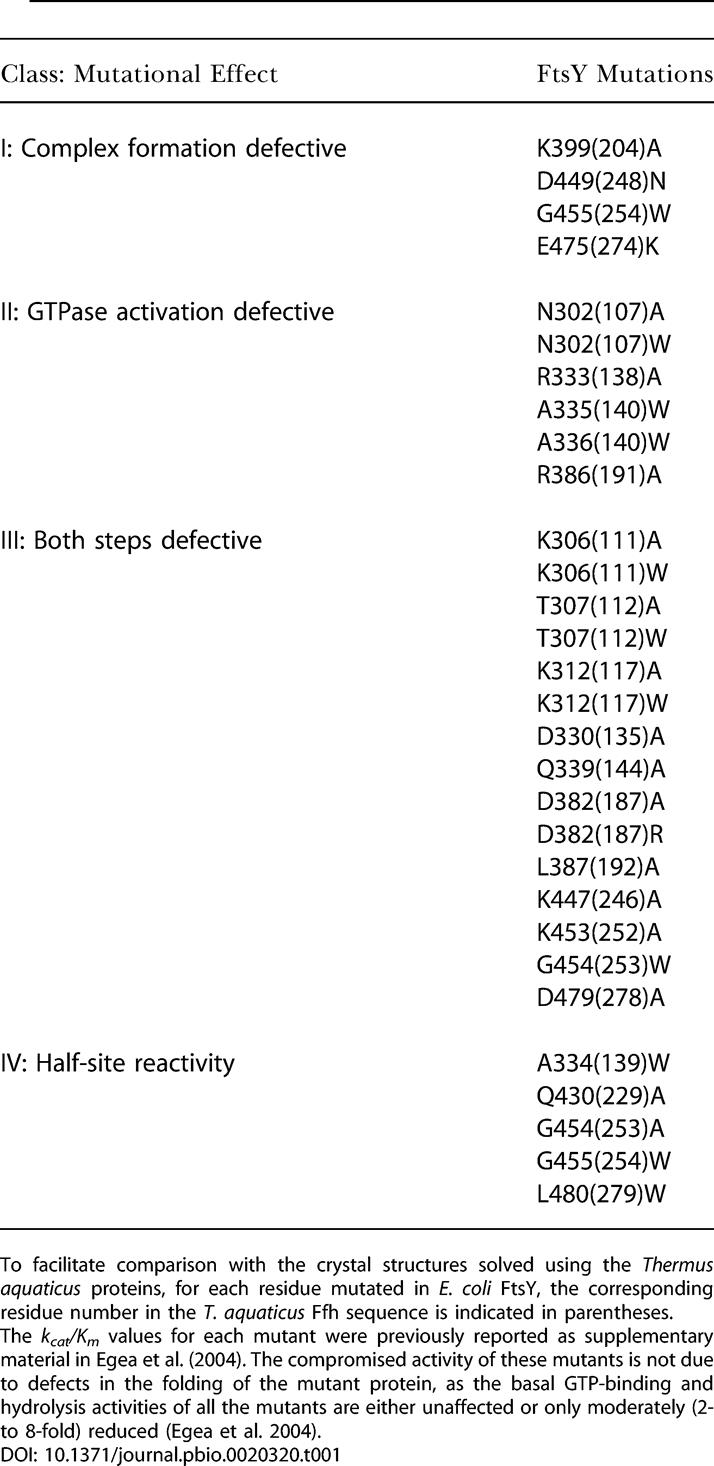
Summary of Different Classes of Mutational Effects

To facilitate comparison with the crystal structures solved using the Thermus aquaticus proteins, for each residue mutated in E. coli FtsY, the corresponding residue number in the T. aquaticus Ffh sequence is indicated in parentheses

The *k_cat_/K_m_* values for each mutant were previously reported as supplementary material in Egea et al. (2004). The compromised activity of these mutants is not due to defects in the folding of the mutant protein, as the basal GTP-binding and hydrolysis activities of all the mutants are either unaffected or only moderately (2- to 8-fold) reduced (Egea et al. 2004)

**Figure 2 pbio-0020320-g002:**
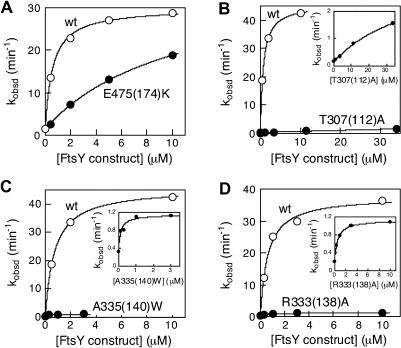
The Effect of FtsY Mutations on the Reciprocally Stimulated GTPase Reaction between Ffh and FtsY The stimulated GTPase reactions of (A) mutant FtsYE475(274)K (•), (B) FtsY T307(112)A (•), (C) FtsYA335(140)W (•), (D) FtsYR333(138)A (•), and wild-type FtsY (○) were determined as described in Materials and Methods. The insets show the reaction curve of the mutant FtsYs on an expanded scale.

All of the Class I–III mutants have deleterious effects on the reciprocally stimulated GTPase reaction between Ffh and FtsY (Figure 2). The protein concentration dependence of this reaction further indicates that the defects in these mutants can be functionally distinguished, allowing us to group them into different classes. For Class I mutants (see Figure 1, blue), the maximal rate of GTP hydrolysis is within 3-fold of that of wild-type FtsY, although a significantly higher concentration of mutant than wild-type FtsY is required to reach saturation (data for a representative mutant are shown in Figure 2A). Thus, these mutants are primarily defective in the Ffh-FtsY complex formation step, but the reciprocal activation of GTP hydrolysis is not significantly affected once the complex is forced to form at the higher FtsY concentrations.

In contrast, for Class II and III mutants, the rate of GTPase reaction remains slow even at saturating concentrations of FtsY (data for representative mutants are shown in Figure 2B–2D). There are two possible explanations for the defects of these mutants: (i) The reciprocal GTPase activation in the Ffh•FtsY complex is compromised, or (ii) both complex formation and reciprocal GTPase activation are affected. The concentration dependence of the stimulated GTPase reaction, however, does not provide an unambiguous way to distinguish between these possibilities, because different steps become rate limiting at different concentration regimes. For wild-type FtsY, the reaction is limited by complex formation with subsaturating FtsY, but becomes limited by GTP hydrolysis with saturating FtsY (Peluso et al. 2001). Thus, for wild-type FtsY the *K_m_* of the reaction (1 μM) does not equal the *K_d_* (16 nM) of the Ffh•FtsY complex. Likewise, the *K_m_* of the reaction with mutant FtsY does not necessarily equal the *K_d_* of the mutant Ffh•FtsY complex, and thus cannot meaningfully distinguish between mutational effects on complex formation and GTPase activation.

**Figure 3:**
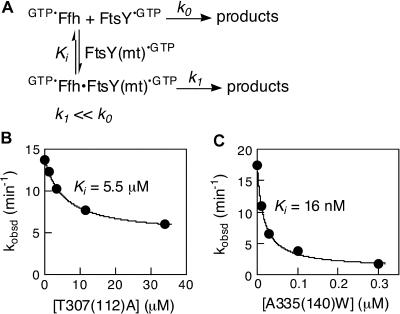
Determination of Complex Formation between Ffh and FtsY Mutants (A) Inhibition assay for determining the affinity of mutant FtsY proteins for Ffh, as described in the text and in Materials and Methods. (B and C) Representative inhibition curves are shown for FtsY mutants (B) T307(112)A and (C) A335(140)W. The data were fit to equation 3 in Materials and Methods.

To circumvent these problems, we devised an assay to determine the ability of each FtsY mutant to inhibit the interaction of wild-type FtsY with Ffh. This assay allowed us to monitor selectively complex formation between Ffh and the FtsY mutants. The conditions of the assay were designed so that in the absence of any mutant FtsY as an inhibitor, a robust GTPase reaction mediated by Ffh and wild-type FtsY was observed (Figure 3A, *k_0_*). Addition of mutant FtsY [FtsY(mt)], which can form a complex with Ffh, will sequester the Ffh molecules into a less active Ffh•FtsY(mt) complex (*k_1_* ≪ *k_0_;* see Figure 2B–[Fig pbio-0020320-g002]D), thus inhibiting the observed GTPase reaction. The reaction was carried out with subsaturating concentrations of wild-type FtsY to ensure that Ffh molecules were predominantly in the free form and able to bind FtsY(mt); under these conditions, the inhibition constant *K_i_* equals *K_d_,* the dissociation constant of the Ffh•FtsY(mt) complex.

Most of the mutants inhibit the reaction only weakly, with inhibition constants about 10^2^-fold weaker than the affinity of wild-type FtsY for Ffh (data for a representative mutant are shown in Figure 3B; a complete list of *K_i_* values is given in Table 2). These mutants are therefore defective in both complex formation and GTPase activation (defined as Class III mutants; see Figure 1, magenta). These mutations involve residues throughout the entire G domain, including the interface between the N and G domains (see Figure 1). Thus, complex formation and GTPase activation are highly coupled. This is presumably due to the fact that the two GTPs are bound at a composite active site formed at the interface, so that many residues that contribute to GTP hydrolysis are also crucial for formation of the interface.

**Table 2:**
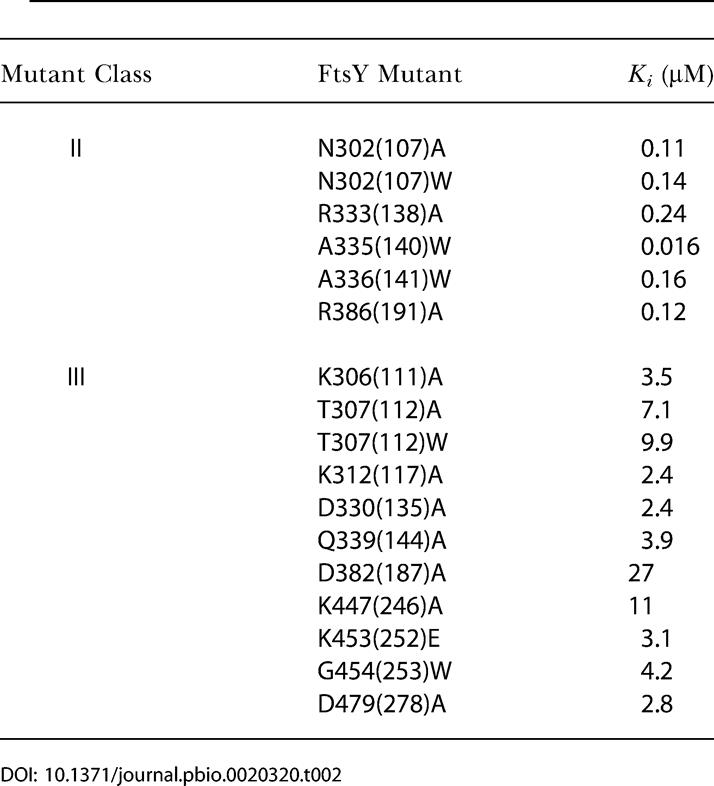
Affinity of FtsY Mutants for Ffh Determined from the Inhibition Assay

**Figure 4:**
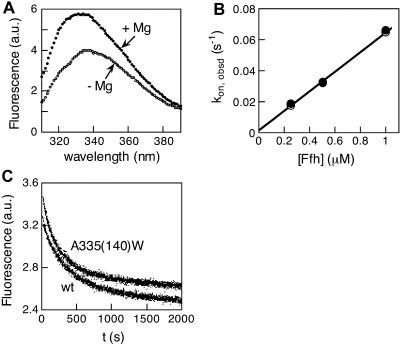
Fluorescence Characterization of Complex Formation between Ffh and Mutant FtsYA335(140)W (A) The tryptophan fluorescence of mutant FtsYA335(140)W changes upon complex formation with Ffh. Complex formation was initiated by the addition of Mg^2+^, as described previously (Shan and Walter 2003). Other Class II mutants do not exhibit as significant a fluorescence change (unpublished data). Thus, the conformational change that alters the environment surrounding the fluorescent W343(148) does not occur even though these mutants can form stable complexes with Ffh. (B) Association rate constants for complex formation with mutant FtsYA335(140)W (○) and wild-type FtsY(•). Linear fits to the data gave association rate constants of 6.36 × 10^4^ and 6.34 × 104 M^−1^ s^−1^ for wild-type and mutant FtsY, respectively. (C) Dissociation rate constants of the Ffh•FtsY complexes formed by mutant FtsYA335(140)W (upper curve) and wild-type FtsY (lower curve). First-order fits to the data gave dissociation rate constants of 3.6 × 10^−3^ and 4.2 × 10^−3^ s^−1^ for wild-type and mutant FtsY, respectively.

In contrast, six mutants (involving mutation of five residues) stood out as strong inhibitors. These mutants (here defined as Class II mutants; see Figure 1, red) can therefore form tight complexes with Ffh and are primarily compromised in the reciprocal activation of GTP hydrolysis. One of these, FtsY A335(140)W, showed an inhibition constant of 16 nM (Figure 3C), indistinguishable from the *K_d_* of the wild-type Ffh•FtsY complex (Peluso et al. 2000). Moreover, the association and dissociation rate constants (Figure 4B and 4C, respectively) for complex formation are also indistinguishable between mutant FtsY A335(140)W and wild-type FtsY, as measured using tryptophan fluorescence changes upon complex formation (Figure 4A) as previously described (Jagath et al. 2000; Peluso et al. 2000). Like the wild-type FtsY, this fluorescence change upon complex formation requires the presence of GTP or the nonhydrolyzable GTP analog GMPPNP (5′-guanylylimidodiphosphate; unpublished data), indicating that the interaction of the Class II mutants with Ffh remains nucleotide dependent. The remaining Class II mutants also have inhibition constants well in the submicromolar range, albeit 10-fold higher than that of FtsY A335(140)W (Table 2).

The mutants described above were identified by analyzing the sum of the two GTP hydrolysis reactions from both Ffh and FtsY. All of the Class II and III mutants must be defective in *both* GTP hydrolysis events; inhibition of only one GTPase site would be predicted to give at most a 2-fold effect because both sites hydrolyze GTP at about the same rate. Half-site mutants defective in GTP hydrolysis in only one active site, however, could be hidden among the Class I and the neutral mutants. To explore this possibility, we monitored the two hydrolysis events individually. To this end, we took advantage of a xanthosine-5′-triphosphate (XTP)-specific Ffh mutant, Ffh D251(248)N. Asp251(248), located in the GTP-binding consensus motif, is conserved throughout the GTPase superfamily and forms a hydrogen bonding network with the N2 and N3 amino protons on the guanine ring (Hwang and Miller 1987; Weijland and Parmeggiani 1993). The Asp → Asn mutation weakens the affinity of Ffh for GTP by 200-fold and increases its affinity for XTP by 10^2^-fold, resulting in a 10^4^-fold switch in nucleotide specificity (SS and PW, unpublished data). In the presence of XTP, Ffh D251(248)N stimulates the GTPase reaction of FtsY and, reciprocally, its XTPase reaction is stimulated by FtsY in the presence of GTP. We therefore used Ffh D251(248)N to monitor the individual hydrolysis events—XTP hydrolysis from Ffh D251(248)N and GTP hydrolysis from the mutant FtsY constructs—in the Ffh D251(248)N•FtsY complex.

**Figure 5:**
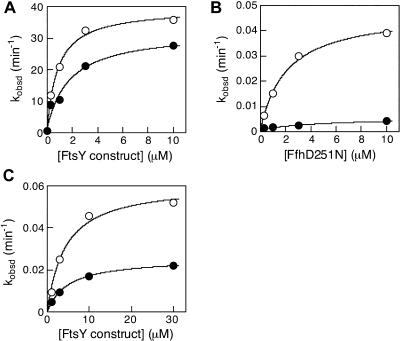
Half-Site Mutants Are Compromised in the Hydrolysis Reaction from the FtsY but Not Ffh Active Site (A) The reciprocally stimulated GTPase reaction with wild-type Ffh for wild-type FtsY (○) and mutant FtsYG455(254)W (•). (B) The FfhD251N-stimulated GTPase reaction from wild-type FtsY (○) and mutant FtsYG455(254)W (•), determined as described in Materials and Methods. (C) The XTP hydrolysis reaction from FfhD251N stimulated by wild-type FtsY (○) and mutant FtsYG455(254)W (•), determined as described in Materials and Methods.

As expected, all of the Class II and Class III mutants were defective in both hydrolysis reactions and, similarly, all but one Class I mutant and most of the neutral mutants showed no significant defect in either of the two reactions (unpublished data). Five half-site mutants, however, stood out from the pool of originally categorized Class I and neutral mutants (see Table 1, Class IV mutants, and Figure 1, green). As expected, the sum of the two GTP hydrolysis reactions was impaired by less than 2-fold in the Class IV mutants (data for a representative mutant are shown in Figure 5A; data for all the Class IV mutants are summarized in Table 3, first column). In contrast, the rates of GTP hydrolysis of all Class IV mutants are reduced by 20- to more than 100-fold (Figure 5B and Table 3, second column). The reciprocal reaction reveals the striking asymmetry of the inhibition: XTP hydrolysis from Ffh D251(248)N is reduced by only 2- to 5-fold (Figure 5C and Table 3, third column).

**Table 3:**
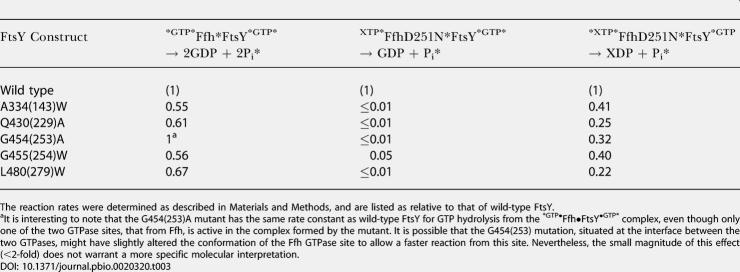
Summary of the Relative Reactivity of Class IV(Half-Site) Mutants in the Individual Nucleotide Hydrolysis Reactions from the Two Active Sites: Reaction of FtsY Mutants with XTP-Specific FfhD251N

The reaction rates were determined as described in Materials and Methods, and are listed as relative to that of wild-type FtsY

^a^It is interesting to note that the G454(253)A mutant has the same rate constant as wild-type FtsY for GTP hydrolysis from the ^*GTP•^Ffh•FtsY^•GTP*^ complex, even though only one of the two GTPase sites, that from Ffh, is active in the complex formed by the mutant. It is possible that the G454(253) mutation, situated at the interface between the two GTPases, might have slightly altered the conformation of the Ffh GTPase site to allow a faster reaction from this site. Nevertheless, the small magnitude of this effect (<2-fold) does not warrant a more specific molecular interpretation

To provide additional evidence for half-site reactivity, we introduced three of the Class IV mutations into an XTP-specific FtsY, FtsY D449(248)N, thereby reversing the nucleotide specificity of the two binding partners. Upon complex formation, FtsY D449(248)N becomes XTP specific and reciprocally activates GTP hydrolysis in Ffh. Consistent with the results observed with Ffh D251(248)N, all Class IV mutations thus analyzed reduce the rate of XTP hydrolysis from mutant FtsYs by more than 10^2^-fold, whereas the reciprocal reaction, GTP hydrolysis by Ffh, is reduced only 2- to 4-fold (Table 4). Thus taken together, Class IV mutations break the symmetry and the remarkable coupling between the two GTPase sites in the Ffh•FtsY complex, such that the nucleotide bound at one active site is hydrolyzed much faster than the nucleotide at the other site.

**Table 4:**
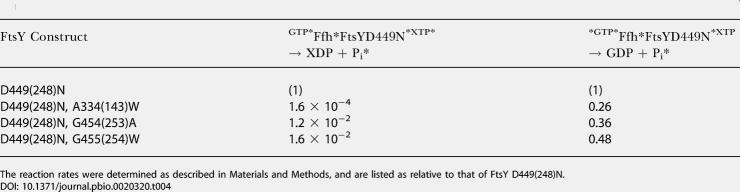
Summary of the Relative Reactivity of Class IV(Half-Site) Mutants in the Individual Nucleotide Hydrolysis Reactions from the Two Active Sites (Continued from Table 3): Reaction of Ffh with Mutant FtsYs that also Bear the XTP-Specific D449(248)N Mutation

The reaction rates were determined as described in Materials and Methods, and are listed as relative to that of FtsY D449(248)N

## Discussion

**Figure 6:**
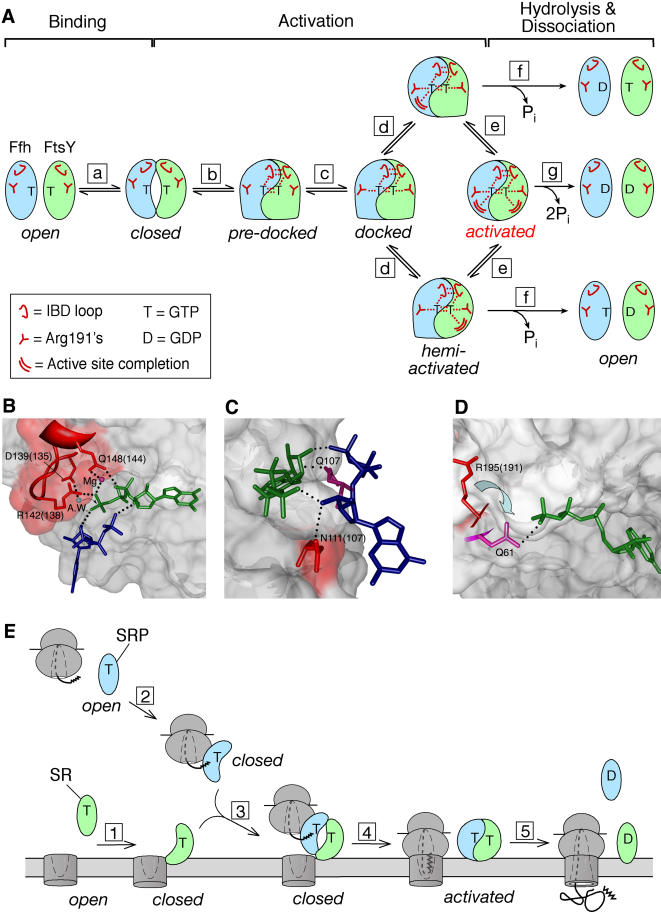
Model for Conformational Changes during Ffh-FtsY Reciprocal GTPase Activation and Implications for the Protein-Targeting Reaction (A) Model for conformational changes during formation of an activated Ffh-FtsY complex. Step a is the rearrangement of both proteins from the open to the closed state during complex formation. Step b is the coordinate docking of the IBD loops into the active sites, and step c is the docking of the Arg191s. Step d is the additional rearrangement of residues that completes one or the other GTPase site. Step e is the rearrangement that completes the other active site. GTP can be hydrolyzed from either the hemiactivated complexes (step f) or the activated complex (step g) to drive complex dissociation. (B–D) Catalytic interactions made by residues exhibiting the Class II phenotype. FtsY is in surface representation, the catalytic residues from FtsY are depicted as red sticks, the nucleotides bound to FtsY and Ffh are in dark green and dark blue, respectively, and the dotted lines depict hydrogen bonds or van der Waals contacts. (B) Interaction of IBD loop with GTP in the FtsY active site. The blue ball represents the attacking water molecule (A.W.); the violet red ball represents the active site Mg^2+^. (C) Interactions of Asn111(107) at the Ffh-FtsY interface. The residue homologous to Asn111, Gln107 in Ffh, is in violet red. (D) Arg195(191) is in a “pending” position. The residue homologous to Arg191 in *Ras,* Gln61, is in violet red. (E) Conformational changes in the GTPase domains of SRP and SR provide potential regulatory points during the protein-targeting reaction. Step 1, SR undergoes an open → closed conformational change upon association with the membrane translocon. Step 2, SRP undergoes an open → closed conformational change upon association with the ribosome and nascent polypeptide. Step 3, complex formation between SRP and SR delivers the cargo to the membrane. Step 4, cargo release from SRP allows the SRP•SR complex to undergo additional conformational changes to activate GTP hydrolysis. Step 5, SRP dissociates from SR after GTP is hydrolyzed. Note that steps 1–3 correspond to Ffh-FtsY binding (step a) in the model shown in (A), step 4 corresponds to Ffh•FtsY activation (steps b–e) in the model shown in (A), and step 5 corresponds to Ffh•FtsY complex dissociation (step g) in the model shown in (A).

The mutational analyses described here define four distinct classes of mutants that map to the Ffh-FtsY interface. Each mutant class blocks the reaction in a different way and at a distinct stage, demonstrating that (i) multiple conformational rearrangements are required to form an activated Ffh•FtsY complex and (ii) some rearrangements can be blocked without preventing other rearrangements from taking place. The different classes of mutant interrupt the reaction in different ways, as represented by the states depicted in Figure 6A, in the pathway of Ffh•FtsY complex formation and reciprocal GTPase activation. The most plausible interpretation of our analysis and the crystallographic analysis of the Ffh•FtsY complex suggest that each of the states blocked by the mutants represents a step on the pathway for the wild-type protein. However, we cannot rule out that some of the rearrangements could occur independently of one another, in which case their depicted order represents only one of the possibilities. Our analysis leads to the conclusion that perturbations, such as those introduced here by site-specific mutations, can modulate specific conformational changes during the Ffh-FtsY interaction. Each of these states provides a potential regulatory point during the protein-targeting reaction, at which analogous effects could be exerted by the cargoes of SRP and SR—the ribosome, signal sequence, and translocon.

### Step A: An “Open”-to-“Closed” Conformational Change upon Complex Formation

We previously showed that FtsY exhibits little discrimination between different nucleotides in its free, uncomplexed form, but gains substantial specificity for GTP only in the Ffh•FtsY complex (Shan and Walter 2003). We therefore proposed that upon complex formation, FtsY changes from a floppy, nonspecific “open” state to a more specific, “closed” state in which the nucleotide is better positioned at the active site and contacts between the guanine ring and Asp449(248), the nucleotide specificity determinant, are established (Figure 6A, step a). The recently determined crystal structures of the Ffh•FtsY complex support this notion (Egea et al. 2004; Focia et al. 2004). Upon complex formation, a major rearrangement occurs at the N-G domain interface, allowing Asp449(248) to move closer to the guanine ring and form hydrogen bonds, thus explaining the enhanced nucleotide specificity of FtsY upon complex formation. We therefore propose that the N-G domain rearrangement during complex formation is central to the open → closed conformational change.

The crystal structure of the Ffh•FtsY complex also shows that Ffh undergoes similar N-G domain rearrangements upon complex formation, although the effects of this rearrangement on nucleotide specificity are less apparent, as free Ffh already displays significant discrimination between nucleotides (SS and PW, unpublished data). Indeed, mutations at the N-G domain interface in either Ffh or FtsY impair complex formation, supporting the functional importance of this rearrangement in both binding partners (Lu et al. 2001). We propose that both free GTPases oscillate between the open and closed states, and that complex formation drives the equilibrium to the closed state (Figure 6A, step a).

### Steps B and C: Docking of Active-Site Residues at the Interface

The Class II mutants allow stable complexes to form but are specifically defective in reciprocal GTPase activation, thus suggesting that the reaction occurs in two steps that can be uncoupled. Further, all of the Class II mutants exhibit significant nucleotide specificity in their interaction with Ffh (unpublished data), suggesting that the mutant proteins have assumed the closed conformation in the complex. Because single mutations in FtsY can disrupt GTPase activation in *both* active sites, the defect in these mutants is not a consequence of simply removing a catalytic residue. Rather, this suggests that even after a stable, closed complex is formed, activation requires additional conformational changes (the “docking” process) that align active-site residues with respect to the bound nucleotides in *both* GTPase sites (Figure 6A, closed →→ docked). Furthermore, as both sites are affected, these rearrangements are highly cooperative and bridge the interface between the two GTPases.

The model in Figure 6 portrays the docking event as two sequential steps: Step b represents the concerted rearrangements of the IBD loops that lead to the predocked state (Egea et al. 2004; Focia et al. 2004). Step c represents the additional rearrangements of the Arg191s in both Ffh and FtsY to form the docked complex. The observation that the crystal structure is “trapped” in a state with the IBD loop docked but with the Arg191s undocked suggests that docking of the IBD loop either precedes that of the Arg191s, as depicted in Figure 6A, or that these two rearrangements can occur independently of one another.

Evidence for the importance of a concerted rearrangement of the IBD loops (step b) comes from three of the Class II mutants, [R333(138)A, A335(140)W, and A336(141)W], which all map to the conserved IBD loop (D^135^TFRAAA). As concluded from the structure, this loop can move relatively independently from the rest of the protein (Egea et al. 2004; Focia et al. 2004). As a result, additional interface contacts are formed between the two loops, and multiple catalytic residues are brought into the active site and positioned close to the nucleotides. Figure 6B highlights the catalytic interactions contributed by these residues: Asp139(135) coordinates the attacking water (A.W.), Arg142(138) coordinates the γ-phosphate oxygen, and Gln148(144) coordinates the β-phosphate oxygen and the active site Mg^2+^. Most importantly, disruption of any of these contacts also destroys activation of the other GTPase site. Therefore, coordinate docking of the IBD loops from *both* interacting partners into their respective active sites is crucial for reciprocal GTPase activation (Figure 6A, step b).

Mutation of Asn302(107) to either Ala or Trp also results in a Class II phenotype. This residue in FtsY hydrogen bonds across the interface to the ribose 3′-OH of the nucleotide bound to Ffh. The ribose 3′-OH reciprocally donates a hydrogen bond back to the γ-phosphate of the twinned substrate in FtsY [Figure 6C, N111(107)]. This interaction is matched by a contact between Q107 of Ffh and the ribose of the nucleotide bound to FtsY (Figure 6C, Q107). These side chains are the only ones that interact with the opposing substrate, and, in addition to the IBD loops, form a second network of catalytically important interactions that bridges the two active sites. Because both of these networks are observed in the crystal structures, we cannot distinguish at this time whether the two networks are assembled coordinately, sequentially, or independently. Potentially, therefore, step b in Figure 6A could be further subdivided.

In contrast to the other Class II mutants, the side chains of the Arg191s point away from the γ-phosphate group in the crystal structure. By analogy to the homologous residue Gln61 in the *Ras*•*Ras*GAP structure, which contacts the γ-phosphate, Focia et al. (2004) proposed that Arg191s are in a “pending” position, forming a “latch” structure that requires additional rearrangements to activate the GTPases, as depicted in Figure 6A (step c) and 6D. The deleterious effect on catalysis displayed by the Arg386(191) mutant strongly supports this notion. Because *both* active sites are affected by the Arg386(191) mutation, the consequences of this additional contact must be transmitted across the interface, perhaps resulting in a slight rearrangement of the twinned GTP molecule to optimize active site-substrate interactions in the other GTPase.

### Step D: Conformational Changes to Activate Individual GTPases

Remarkably, the Class IV, or half-site, mutants demonstrate that activation of the individual GTPase sites can be further uncoupled from one another. This suggests that after all the molecular rearrangements required to activate the interacting GTPase have been accomplished, additional rearrangements are required to complete each active site. Further, these rearrangements either occur late in the docking process (Figure 6A) or they occur independently of the various docking steps. In contrast to the docking steps that are tightly coupled between the two active sites, these additional rearrangements can occur independently in one GTPase but not the other, leading to the formation of “hemiactivated” intermediates (Figure 6A, step d).

Four of the five Class IV half-site mutants (see Figure 1, green) are positioned away from the γ-phosphate group: G454(253) and G455(254) map to the conserved DARGG motif at the NG domain interface, L480(279) maps to the “closing loop” that packs against the guanine base, and Q430(229) is situated away from any residue for which a function can be assigned intuitively. The mechanistic interpretation of these mutants will have to await structural information from crystallization of the mutant proteins and additional characterization of the dynamics during Ffh-FtsY association and activation.

Importantly, all of the half-site mutants are less than 2-fold reduced in the rate of multiple-turnover GTPase reactions, indicating that multiple cycles of Ffh•FtsY complex formation and dissociation can still occur efficiently. Thus, only one of the two bound GTPs needs to be hydrolyzed in order for the Ffh•FtsY complex to dissociate (Figure 6A, step f). In the wild-type Ffh•FtsY complex, it is well established that both nucleotides are hydrolyzed during each turnover. Thus, after a hemiactivated state is formed, rearrangement of the other GTPase site must follow on a time scale faster than the rate of GTP hydrolysis or complex dissociation (Figure 6A, step e), so that a fully activated complex is formed and both GTP molecules are hydrolyzed (Figure 6A, step g).

### Implications for the Protein-Targeting Reaction

During protein targeting, SRP and SR are thought to interact with their respective cargoes, the RNC and the translocon. Thus, targeting involves a series of ordered steps in which cargo binding and release must occur at the proper stages. Each of the conformational changes in the GTPase domains of SRP and SR described above provides a potential point at which such control can be exerted, thereby coordinating the loading and unloading of cargoes (Figure 6E).

One possible view is that the switch from open to closed conformation provides the regulatory point that distinguishes free from cargo-loaded SRP and SR. Under cellular conditions, both SRP and SR are likely to be GTP bound before entering the targeting reaction. Thus, GTP binding per se cannot be the switch that sets these GTPases to the “on” state, as happens with classical signaling GTPases. Free SRP receptor is predominantly in the open conformation; interaction with phospholipid membranes and the translocon could shift its conformational equilibrium towards the closed state (Figure 6E, step 1), thereby facilitating its interaction with SRP. Reciprocally, the SRP could undergo a similar open-to-closed conformational change, facilitated by association with the RNC (Figure 6E, step 2). In this view, the SRP and receptor molecules that are prebound to their respective cargoes are “primed” to interact with each other, ensuring efficient delivery of cargo proteins to the membrane and avoiding futile cycles of SRP-receptor interactions (Figure 6E, step 3).

Once at the membrane, it is crucial that SRP releases its cargo to the translocon before it dissociates from the SRP receptor. Because both GTPases reciprocally activate each other, regulation of GTP hydrolysis must involve mechanisms different from regulation by external GAPs, as happens with classical signaling GTPases. The conformational changes required for GTPase activation (Figure 6A, steps b–d) provide the potential to control the relative timing of the cargo release versus GTP hydrolysis steps. In solution, the SRP•SR complex exists only transiently, with a half-life of less than 1 s, because rapid GTP hydrolysis drives complex dissociation. However, RNC, SRP, and SR can be cross-linked to each other in the absence of the translocon (Song et al. 2000). Although this does not provide conclusive evidence, it is an attractive possibility that RNC could delay GTP hydrolysis, possibly by inhibiting one of the docking steps described here (Figure 6A, steps b–d), thereby ensuring that the cargo is released from SRP before GTP is hydrolyzed (Figure 6E, step 4). Release of the cargo then allows the SRP•SR complex to undergo the additional rearrangements to activate GTP hydrolysis, leading to complex dissociation (Figure 6E, step 5).

The demonstration that hemiactivated complexes can exist and that hydrolysis of a single GTP is sufficient for complex dissociation (Figure 6A, steps d and f) raises intriguing questions as to the precise role of the individual GTP hydrolysis events during each cycle of the targeting reaction. Potentially, asymmetric, half-site hydrolysis could be used to introduce branches into the pathway, leading to abortive targeting reactions. In this way, the GTP hydrolysis events could have proofreading roles similar to those proposed for translation elongation factors to help ensure the fidelity of protein targeting. The analysis described here therefore not only dissects the reciprocal GTPase activation events into a set of conformational rearrangements, but also provides invaluable tools to assess the role of these states as potential control points in the targeting reaction.

## Materials and Methods

### 

#### Cloning and purification of mutant proteins.

Expression plasmids for mutant FtsYs were constructed from that for wild-type FtsY(47–497) using the QuickChange Mutagenesis protocol (Stratagene, La Jolla, California, United States). Mutant FtsY proteins were expressed and purified using the same procedure as that for wild-type FtsY (Powers and Walter 1997; Peluso et al. 2001).

#### Kinetics.

GTP hydrolysis reactions were followed and analyzed as described in Peluso et al. (2001). The reciprocally stimulated GTPase reactions between Ffh and FtsY were measured in multiple-turnover experiments ([GTP] > [E]) with a small fixed amount of Ffh and varying amounts of wild-type or mutant FtsY, and the FtsY concentration dependences were analyzed as described in Peluso et al. (2001).

The Ffh(D251N)-stimulated GTPase reaction of FtsY was determined in single-turnover experiments in the presence of 10 μM FtsY and varying amounts of Ffh(D251N), with 50 μM XTP present to selectively occupy the Ffh(D251N) active site [K^XTP^_d_ = 0.37 and 460 μM for Ffh(D251N) and FtsY, respectively; SS and PW, unpublished data; Shan and Walter 2003]. The concentration dependence of the observed GTPase rate constant is fit to equation 1, in which *k_max_* is the maximal rate constant with saturating Ffh(D251N), and *K_1/2_* is the concentration of Ffh(D251N) required to reach half saturation.



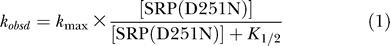



The reciprocal reaction, the FtsY-stimulated XTPase reaction of Ffh(D251N), was determined in single-turnover experiments with 1 μM Ffh(D251N) and varying amounts of FtsY. 50 μM GTP was present to ensure that FtsY was selectively bound with GTP [K^GTP^_d_ = 15 μM and 101 μM for FtsY and Ffh(D251N), respectively; SS and PW, unpublished data; Shan and Walter 2003]. The FtsY concentration dependence is fit to equation 2, in which *k_max_* is the maximal rate constant with saturating FtsY, and *K_1/2_* is the concentration of FtsY required to reach half saturation.



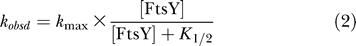



The affinity of mutant FtsY proteins for Ffh was determined using an inhibition assay that measures the ability of mutant FtsYs [FtsY(mt)] to inhibit the interaction between Ffh and wild-type FtsY, as described in detail in the text (see Figure 3A). The data are fit to equation 3, derived from Figure 3A.



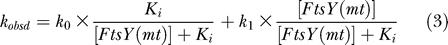



#### Fluorescence measurements.

Fluorescence emission spectra were acquired as described in Peluso et al. (2000) in the presence of 1 μM mutant or wild-type FtsY, 2 μM SRP, and 100 μM GppNHp, and complex formation was initiated by addition of Mg^2+^ as described in Shan and Walter (2003). The rate constants for association and dissociation of the Ffh•FtsY complex were determined by following the time course of the fluorescence change at 335 nm as described in Peluso et al. (2000, 2001).

## Abbreviations

ER: endoplasmic reticulum
GAP: GTPase-activating protein
GEF: guanine nucleotide exchange factor
GMPPNP: 5′-guanylylimidodiphosphate
RNC: ribosome•nascent chain complex
SR: SRP receptor
SRP: signal recognition particle
XTP: xanthosine-5′-triphosphate
